# Biomedical Teleacupuncture between China and Austria Using Heart Rate Variability, Part 1: Poststroke Patients

**DOI:** 10.1155/2011/782489

**Published:** 2011-06-09

**Authors:** Lu Wang, Jan Valentini, Kazuo Sugimoto, Weiping Cheng, Guangyu Cheng, Haoming Geng, Ingrid Gaischek, Haixue Kuang, Gerhard Litscher

**Affiliations:** ^1^Research Unit of Biomedical Engineering in Anesthesia and Intensive Care Medicine, TCM Research Center Graz, Medical University of Graz, 8036 Graz, Austria; ^2^Heilongjiang University of Chinese Medicine, Harbin 150040, China

## Abstract

*Background*. Acupuncture has been reported to affect the human autonomic system. Within this pilot study, teleacupuncture between China and Austria is used for the first time for quantifying the effects of heart rate variability (HRV) in poststroke rehabilitation. *Methods*. In 29 Chinese post-stroke patients (15 f, 14 m; mean age ± SD 64.7 ± 11.3 years; range 40–80 years) electrocardiographic signals before, during, and after acupuncture at the acupoint Tongli (HT 5) were recorded in Harbin and analyzed in Graz using teleacupuncture via internet. HRV data were analyzed in the time and frequency domain, and a protocol from Austria was sent to the team in China immediately after the treatment and recording session. *Results*. Acupuncture does not change heart rate in the post-stroke patients; however, total HRV increased significantly (*P* < .05) during and 5–10 minutes after acupuncture. In addition, balance between sympathetic and parasympathetic activity (low frequency/high frequency HRV ratio) changes markedly during treatment. *Conclusions*. Based on innovative HRV analysis, it could be demonstrated that teleacupuncture between China/Harbin and Austria/Graz over a distance of about 8,500 km is no longer a future vision; it has become reality.

## 1. Introduction

In recent times, sophisticated medical equipment has become the main reason and factor in the advancement of acupuncture. However, many hospitals for Traditional Chinese Medicine (TCM) in China do not have clinical engineering departments. The establishment of *teleacupuncture* is an excellent new possibility for effectively combining transcontinental clinical and basic acupuncture research [1–4].

The novel concept of the current teleacupuncture technology has been implemented at the TCM Research Center Graz in Austria (http://litscher.info and http://tcm-graz.at) in 2010 in cooperation with the Heilongjiang University of Chinese Medicine in Harbin, China, over a distance of about 8,500 km.

This paper describes the first results from teleacupuncture measurements in poststroke patients using computer-based heart rate variability (HRV) recordings before, during, and after acupuncture under standardized clinical conditions in China. Analyses were performed in Graz, Austria, immediately after acupuncture sessions, and the protocol was then sent back to Harbin, China, via internet.

## 2. Subjects and Methods

### 2.1. Patients

Electrocardiographic signals were recorded in 29 adult patients (15 female, 14 male; mean age ± SD 64.7 ± 11.3 years; range 40–80 years). All patients with the Chinese diagnosis “Zhong Feng” received acupuncture treatment for poststroke rehabilitation and were not under the influence of centrally active medication. None of the patients had a body temperature out of the normal range (mean ± SD 36.2 ± 0.2°C; range 36.0–36.7°C). Body height was 165.2 ± 8.1 cm, and body weight was 64.3 ± 9.5 kg. The study design was approved by the local ethics committee, and the noninvasive recording procedure (electrocardiogram (ECG)) was performed in compliance with the Declaration of Helsinki. All persons were informed about the nature of the investigation and the results of the own measurement, at the latest one day after ECG-monitored acupuncture session.

### 2.2. Biosignal Recording and Evaluation Parameters

Basis for determining HRV is the duration of RR intervals measured during a special time period (5 minutes). ECG registration was performed with three adhesive electrodes (Skintact Premier F-55; Leonhard Lang GmbH, Innsbruck, Austria) applied to the chest. 

**Figure 1:**
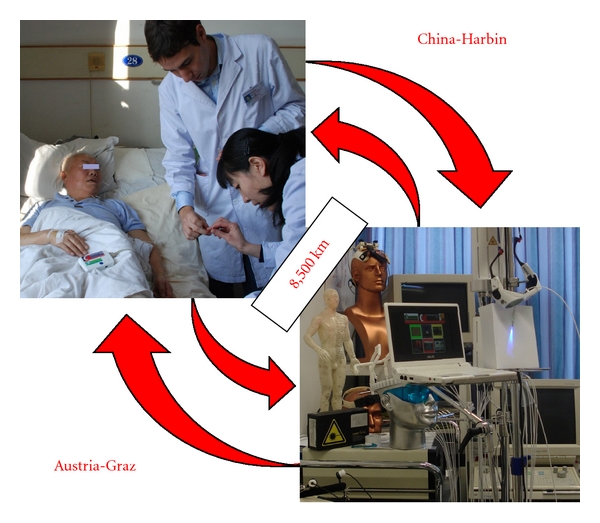
Teleacupuncture between China and Austria. Left: acupuncture research in a poststroke patient at the Heilongjiang University of Chinese Medicine using equipment from the Medical University of Graz (with permission of the patient and the two doctors from Graz and Harbin). Right: data analysis in the high-tech acupuncture lab at the Medical University in Graz, Austria.

For our investigations at the Heilongjiang University of Chinese Medicine, a medilog AR12 HRV (Huntleigh Healthcare, Cardiff, United Kingdom) system which was partly developed in Austria was used (see Figure 1, left side). By recording with 4096 samples per second, the new system can detect *R *waves extremely accurately. *R*-peak time resolution is 244 microseconds and the *P* and *T* time resolution 1,953 microseconds. The dimensions of the HRV recorder are 70 × 100 × 22 millimeters, the weight is about 95 grams with batteries [5].

All raw data were stored digitally on a special memory card. After removing the card from the portable system, the data were read by a card reader connected with a standard computer in China and then transferred to the TCM Research Center Graz via internet. With a new software [1, 2, 5], the biosignals were analyzed, and HRV was displayed in a way to help to judge the function of the autonomic nervous system. Viewing this innovative kind of analysis helps to show how well the human body reacts to sport, stress, recovery, and also acupuncture [1–5]. For offline visual inspection, all ECG raw data can be displayed on a screen.

Mean heart rate (HR), total HRV, and the LF (low frequency)/HF (high frequency) ratio of HRV served as evaluation parameters. These parameters are recommended by the task force of the European Society of Cardiology and the North American Society of Pacing and Electrophysiology [6] and provide an understanding of the effects of sympathetic and parasympathetic systems on HRV. In addition, the blood pressure was recorded discontinuously.

### 2.3. Acupuncture and Procedure

**Figure 2:**
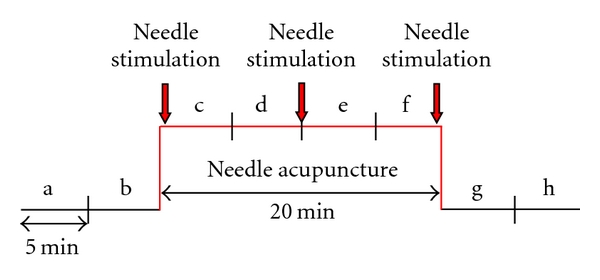
Measurement procedure. The data before (measurement phases a, b), during (c–f), and after (g, h) manual needle acupuncture stimulation were measured and statistically analyzed.

Acupuncture was performed at the acupoint Tongli (HT.5) on the left heart meridian in all patients. Tongli is located 1 cun (relative body measure; the breadth of the distal phalanx of the thumb) proximal to HT.7 (Shenmen), radial to the tendon of m. flexor carpi ulnaris. Its use is also indicated in cases of speech disturbances, aphasia, and mental disorders [7]. Sterile single-use needles (0.30 × 30 mm; Huan Qiu, Suzhou, China) were used. Needling was performed perpendicularly (depth about 1 cm), and the needles were stimulated clockwise and counterclockwise for 15 seconds each, with two rotations per second, resulting in 30 rotations per stimulation. Stimulation was done immediately after inserting the needle, 10 minutes later, and before removing the needle (comp.  Figure 2). For the present study, an acupuncture session between the third and seventh treatment was chosen for ECG monitoring.

### 2.4. Statistical Analysis

Data were analyzed using SigmaPlot 11.0 software (Systat Software Inc., Chicago, USA). Graphical presentation of results uses box plot illustrations. Testing was performed with Friedman repeated measures ANOVA on ranks and Tukey test. The criterion for significance was *P* < .05.

## 3. Results

**Figure 3:**
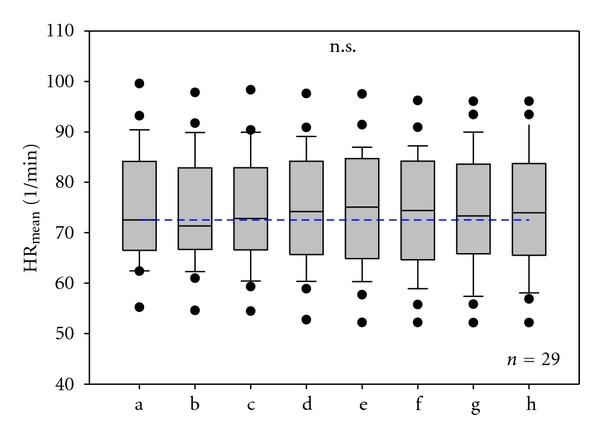
Mean heart rate. Box plot illustration in 29 poststroke patients before (a, b), during (c–f), and after (g, h) needle acupuncture. No significant (n.s.) changes were found. The horizontal line in the box gives the position of the median. The end of the box defines the 25th and 75th percentile, and the error bars mark the 10th and 90th percentile.

Results of mean HR from the ECG recordings before, during, and after acupuncture of the 29 patients are shown in Figure 3. There were no significant alterations within the different conditions (a–h). Blood pressure, measured discontinuously, did not change significantly during the measurement procedure.

**Figure 4:**
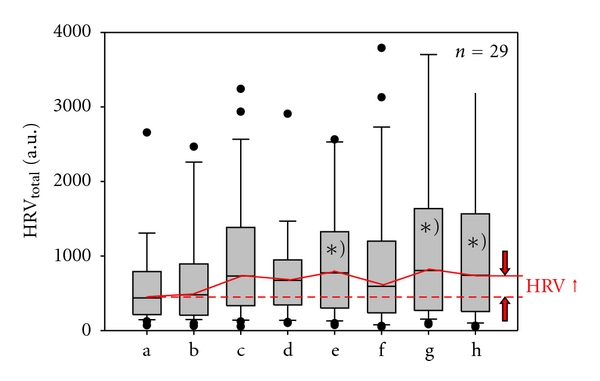
Total heart rate variability. Graphical box plot presentation of significant (^∗)^ *P* < .05) changes during (e) and after (g, h) acupuncture. Note the significant increase in total HRV. For further explanations compare withFigure 3.

Analysis of total HRV showed the following interesting results (Figure 4). There was a positive effect in the sense of a significant increase in total HRV during and after acupuncture. This effect appeared already after inserting the needles (phase c) and reached a significant level after the second stimulation period (phase e). Then, while removing the needles, a slight decrease of HRV could be observed. After the third stimulation and removing the needles, there was again a significant increase in HRV (phases g and h).

**Figure 5:**
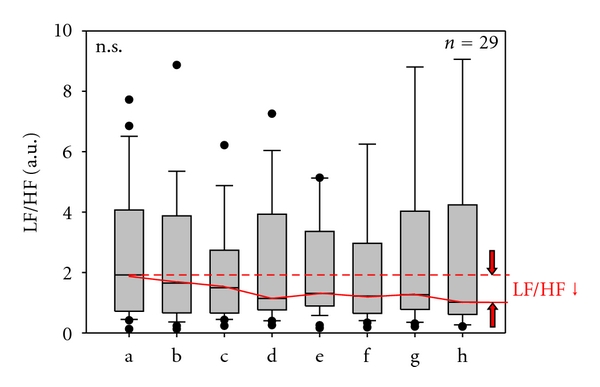
LF (low frequency)/HF (high frequency) ratio. Note that the ratio decreases during acupuncture treatment in 29 poststroke patients. For further explanations, see Figure 3.

Furthermore, the biosignal monitoring during acupuncture showed substantial reductions in the LF/HF ratio (Figure 5). During acupuncture, the values decreased markedly and reached a maximal reduction 5 minutes after removing the needles (phase h), even though the magnitude of the changes varied from patient to patient.

## 4. Discussion

Since the pioneering work of the TCM Research Center at the Medical University of Graz in Austria, high-tech acupuncture including teleacupuncture has been recognised as a unique and powerful tool for investigating effects of acupuncture, especially within transcontinental studies [1–4, 8].

There have been several other experiments with telemedicine in China, and other telemedical services are in operation [9]. Other authors stated in a publication that “China is a large country and is rapidly modernizing. In reporting about telemedicine in China as well as reporting about all technology associated with computer and communication applications in China, one can never be sure whether one's information is up to date, complete and accurate” [9]. To the best of our knowledge, teleacupuncture has not previously been reported and was first described by our research group last year [1–4, 8, 10]. It is defined as the exchange of medical acupuncture information across distance [10]. The realization of this innovative concept of teleacupuncture could probably be useful under special circumstances (cooperation between experts from different continents) as demonstrated in our Sino-Austrian collaboration [8].

Within the present preliminary clinical pilot study, teleacupuncture using data analysis of different parameters like HR and HRV in the time and frequency domain was performed for control of possible therapeutic effects of acupuncture in a collective of poststroke patients in Harbin. The acupuncturists in China were informed about the findings immediately after the analysis in Graz, and the effects of the therapy could be demonstrated objectively.

HRV is a widely accepted measurement method for the assessment of the patient's neurophysiological state. The better HRV, the healthier the patient. Patients at risk for special diseases like hypertension, arteriosclerosis, diabetes, cancer, or other health problems have low HRV. However, even today the quantification of HRV and the knowledge of the mechanism as well as the measurement in special diseases still pose unresolved questions. In general, the RR intervals in the ECG are controlled by the blood pressure control system, influenced by the hypothalamus and in particular controlled by the vagal cardiovascular center in the lower brainstem. HRV can be quantified over time using registration of percentage changes in RR-intervals in the time domain as well as the changes in the frequency range by analysis of electrocardiographic power spectra [6]. HRV is interpreted as a brainstem reflex with an afferent arc via the vagus and glossopharyngeal nerves and an efferent arc mainly via vagal fibres [11, 12]. HRV has stochastic and rhythmic components. With spectral analysis, variability can be classified into individual ranges which represent biological rhythms. The following influences can be distinguished for different ranges of HRV: (a) respiratory sinus arrhythmia (approximately 0.15–0.5 Hz); centrally nervous respiratory impulses and interaction with pulmonary afferents; (b) the so-called “10-s-rhythm” (approximately 0.05–0.15 Hz); natural rhythm of cardiovascularly active neurons in the lower brainstem (circulatory center and its modulation by feedback with natural vasomotoric rhythms via baroreceptor feedback); (c) longer wave HRV rhythms (approximately <0.05 Hz); effects from the rennin angiotensin system or temperature regulation as well as metabolic processes [6].

The scope of HRV is not yet completely clear, but it is known that there are intraindividual and interindividual variances and that HRV depends on age, circadian variations (sleep-wake-cycle), physical condition, and mental and physical exertion. HRV can also be affected by diverse conditions such as age-related diseases (diabetic neuropathy, renal failure, essential hypertension, cardiac disorders, coronary artery disease, and intracranial lesions) and different medications. The narrowness of HRV after heart transplantation [13] is similar to that seen in deep comatose patients and in brain-dead subjects [12], in whom complex reflex mechanisms are no longer generated or regulated in the brain. In contrast, heart transplantation totally interrupts peripheral autonomic afferences and efferences.

Within this study, patients after stroke with the Chinese diagnosis “Zhong Feng” were investigated. This diagnosis means that all patients had wind stroke (Zhong Feng) attacking the channels and collaterals caused by increased liver yang and kidney yin and yang deficiency. 

As already mentioned, HRV can be used as reliable indicator of the state of health [10]. It becomes less random with the aging process and the appearance of age-related diseases [14, 15]. However, it could be demonstrated that in special syndromes like fatigue and stress one can counteract this process using different preventive methods like acupuncture [10, 16]. This has been demonstrated in recent investigations concerning patients with burn-out syndrome as performed in a further teleacupuncture study between Beijing and Graz [1, 10].

The main findings of this study in poststroke patients were that acupuncture has a pronounced effect on HRV in the sense of an increase while at the same time HR did not change. Balance between sympathetic and parasympathetic activity, expressed by the LF/HF ratio, decreased. The interpretation of these results is not very easy. Li et al. [17], for example, reported that a state of fatigue can significantly influence HRV (reduced LF/HF ratio) after acupuncture, while persons not being fatigued showed no changes. Arai et al. [18] also found increased vagal activity (reduced LF/HF ratio) after acupressure. A systematic clinical review on acupuncture and HRV published recently in 2010 [19] searched the literature using 14 data bases without language restrictions. Twelve randomized clinical trials (RCTs) met all inclusion criteria. Five RCTs found significant differences in HRV between patients treated with acupuncture versus those treated with sham acupuncture (controls). The majority of the other RCTs showed inconsistent results [19]. The authors from Korea and the United Kingdom stated that more rigorous research appears to be warranted. The number, size, and quality of the RCTs that are available are too low to draw firm conclusions.

The same research team also published a systematic review on the topic “acupuncture for functional recovery after stroke” in November 2010 [20] and one article concerning “acupuncture in the rehabilitation of poststroke hemiplegic patients” [21]. Both reviews came to the conclusion that there is limited evidence for a positive effect of acupuncture as treatment for functional recovery after stroke.

It has to be mentioned that our pilot study has some limitations, both from the Western point of view (no RCT) and from the TCM viewpoint (no personalized acupuncture scheme). However, it clearly shows that the new methodological procedure of teleacupuncture can produce important additional clinical references that acupuncture has positive effects on HRV and therewith on the state of health [10, 11].

The following conclusion can be drawn from our preliminary teleacupuncture study.

Firstly, acupuncture at the acupoint Tongli (HT 5) does not change HR in poststroke patients; however, total HRV was increased significantly during and 5–10 minutes after acupuncture.

Secondly, the balance between sympathetic and parasympathetic activity (LF/HF ratio reduction) changed markedly during and after manual needle acupuncture.

Thirdly, we have shown that teleacupuncture over a distance of about 8,500 km is no longer a future vision; it has already become reality between China and Austria.

##  Funding

This work was supported by the Austrian Federal Ministries of Science and Research and of Health and the Eurasia Pacific Uninet (project “Bioengineering and clinical assessment of high-tech acupuncture—a Sino-Austrian research pilot study”); and the OeNB Jubiläumsfonds (Project no. 13463) and performed within the areas “Sustainable Health Research” and “Neuroscience” at the Medical University of Graz.
